# Henoch-Schönlein Purpura Presenting as Severe Gastrointestinal and Renal Involvement with Mixed Outcomes in an Adult Patient

**DOI:** 10.7759/cureus.1088

**Published:** 2017-03-09

**Authors:** Raj Shah, Madhuri Ramakrishnan, Alexis Vollmar, Amanda Harrell, Richard Van Trump, Amgad Masoud

**Affiliations:** 1 Department of Internal Medicine, University of Missouri - Kansas City; 2 Medical Student, University of Missouri; 3 Internal Medicine, Freeman Hospital; 4 Anesthesiology, University of Missouri - Kansas City

**Keywords:** henoch-schönlein purpura, iga vasculitis, end stage renal disease, male, plasmapheresis, immunosuppression, adult

## Abstract

Henoch-Schönlein purpura (HSP) is typically seen as a self-limiting disease in children, but can present more severely in adults, especially when there is renal involvement. Management of HSP in adults also remains a controversial topic with very few studies evaluating available therapies. In this case, HSP presenting as a combination of severe gastrointestinal involvement and a rapid decline in renal function in an adult patient directed our therapy.

The patient was a 48-year-old Caucasian male with no known past medical history, who presented with a combination of purpuric rash over the lower extremities, severe abdominal pain with upper gastrointestinal bleeding and a rapidly increasing serum creatinine, with hematuria. He initially underwent a skin biopsy, along with investigation for other possible causes, including autoimmune and infectious etiologies, which were negative. He was started on therapy for presumed HSP with intravenous methylprednisolone. The skin biopsy, however, was not conclusive, and the patient had no improvement in his clinical status. He then underwent a kidney biopsy that was consistent with HSP nephritis (immunoglobulin A (IgA) predominant glomerulonephritis with crescents), and esophagogastroduodenoscopy (EGD) that showed mucosal inflammation, ulcerations, and stigmata of bleeding—findings that were consistent with ischemia. Cyclophosphamide was added to the regimen at this time. However, he had worsening abdominal pain, continued gastrointestinal bleeding, now with hematochezia, and also worsening renal function that required dialysis. Plasmapheresis was then initiated on days alternating with dialysis. This resulted in the improvement of his gastrointestinal symptoms, but no recovery was seen of his renal function, and the patient required outpatient dialysis.

This case report exhibits the unique presentation of severe gastrointestinal (GI) manifestations and rapid progression to renal failure in an adult patient with partial resolution of his severe manifestation after therapy was escalated as above. There was no established protocol that guided this therapy, which reflects the need for more studies on adult HSP.

## Introduction

Henoch-Schönlein purpura (HSP), also commonly called immunoglobulin A (IgA) vasculitis, is a small vessel vasculitis, that usually presents with the classic symptoms of purpura, arthritis/arthralgia, abdominal pain, and renal disease [1]. It is seen primarily in children, with an estimated annual incidence of 15 cases/ 100,000 [2-3]. In contrast, it is a rare disease entity in adults, with an estimated annual incidence of 1.3 cases/100,000 [3-4]. As such, the disease is less well studied in adults, and much less is known about its natural history. The disease tends to be self-limited in children [2-3], but older patients exhibit more severe clinical features [3-4]. Very little is also known about the management in adults, as there are very few studies evaluating the efficacy of the different treatment options, including steroids, immunosuppressive drugs, and plasmapheresis.

In this report, we review a case of HSP presenting with severe gastrointestinal and renal involvement in a 48-year-old Caucasian male with no previous medical history, with mixed outcomes associated with the aggressive therapy we utilized. Informed consent was obtained from the patient for this study.

## Case presentation

A 48-year-old Caucasian male with no past medical or surgical history (except for a 20-pack-year smoking history) initially presented with a new onset rash of one day duration covering his lower extremities, with accompanying swelling and soreness of his hands and feet. An examination revealed stable vital signs, purpura along with some pustular lesions scattered over both lower extremities and suprapubic region, and bilateral feet and hand swelling. Urine studies were remarkable for urine protein of 203 mg/dl, 5–10 red blood cells per high power field, and 2–5 hyaline casts per field. The urine protein: creatinine ratio was 859 mg/g. Creatinine at that time was 1.6 mg/dL. There was concern for HSP, and a skin biopsy of his lower extremity was obtained. The patient was discharged with the results of the biopsy pending.

Five days later, he presented to the emergency department after developing coffee-ground emesis, melena, and diffuse abdominal pain. He continued to have the purpuric rash over his lower extremities. Laboratory studies revealed a worsening creatinine of 2.4 mg/dL, a serum albumin of 1.6 mg/dL, and an elevated C-reactive protein of 5.3 mg/dL. It also revealed worsening hematuria (now 25–50 red blood cells per high power field) and proteinuria (now 9 g/g). The patient underwent workup for other possible vasculitis and glomerulonephritis, with labs including antinuclear antibody (ANA), perinuclear anti-neutrophil cytoplasmic antibodies (p-ANCA), cytoplasmic antineutrophil cytoplasmic antibodies (c-ANCA), anti-glomerular basement membrane (anti-GBM), complement levels, cryoglobulins, human immunodeficiency virus (HIV), hepatitis panel, IgA levels, and serum protein electrophoresis, all of which were within normal limits. His hemoglobin, which was 15.5 g/dL on presentation, trended down to 11 g/dL over two days.

After consulting Rheumatology, empiric therapy of pulse IV methylprednisolone 500 mg daily for three days was initiated, with continuation of 60 mg intravenous (IV) twice daily. His renal function continued to deteriorate, with decreasing urine output and rising creatinine, now up to 3.70 mg/dL. The skin biopsy results showed dermal neutrophils, eosinophils, and extravasation of red blood cells, which was not definitive for HSP.

**Figure 1:**
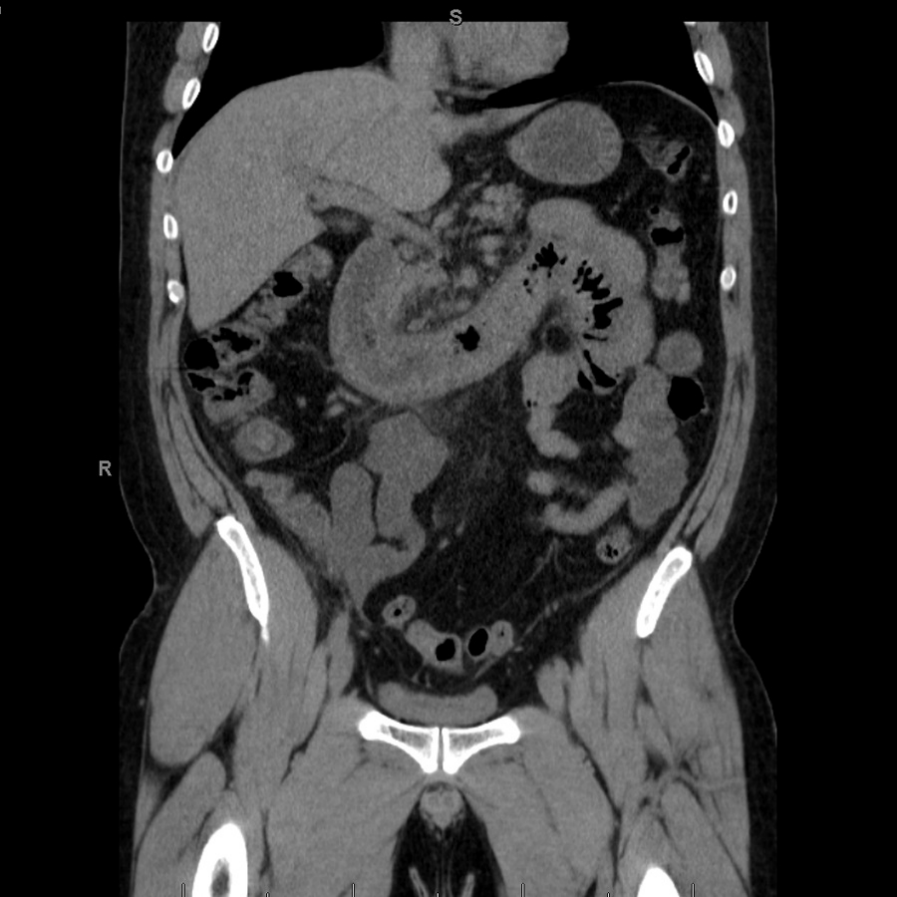
Coronal section of CT abdomen and pelvis with contrast showing bowel wall inflammation and edema.

With no improvement of gastrointestinal or renal manifestations and no definitive diagnosis yet, he was sent for a renal biopsy and was started on hemodialysis three times a week. He also underwent an esophagogastroduodenoscopy that showed Grade B esophagitis with > 5 mm long mucosal break in the lower esophagus and severe patchy inflammation, erythema, edema, petechiae, ulceration, and friability in the duodenum. There were stigmata of recent mucosal bleeding and the findings were highly suggestive of ischemia. Sigmoidoscopy showed continual, patchy inflammation and erythema from the rectum to the sigmoid colon. These findings were corroborated by a computed tomography (CT) scan of the abdomen and pelvis that showed severe pan-inflammation of the duodenum and colon with thickening of the small and large bowel walls and multiple dilated bowel loops throughout. It also showed nonspecific periappendiceal inflammation and dilation of the distal tip with accompanying ascites [Figure 1]. A surgical consult was requested given the above findings, and no surgical intervention was recommended at the time.

**Figure 2:**
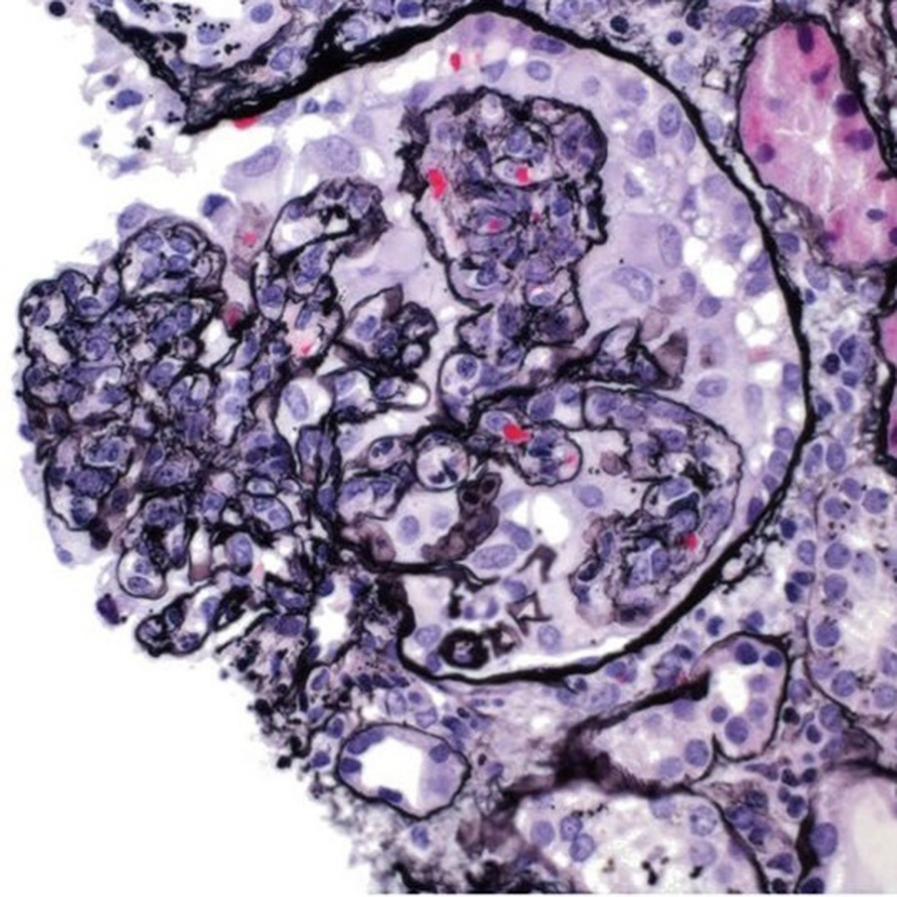
Light microscopy of kidney biopsy showing crescents.

**Figure 3:**
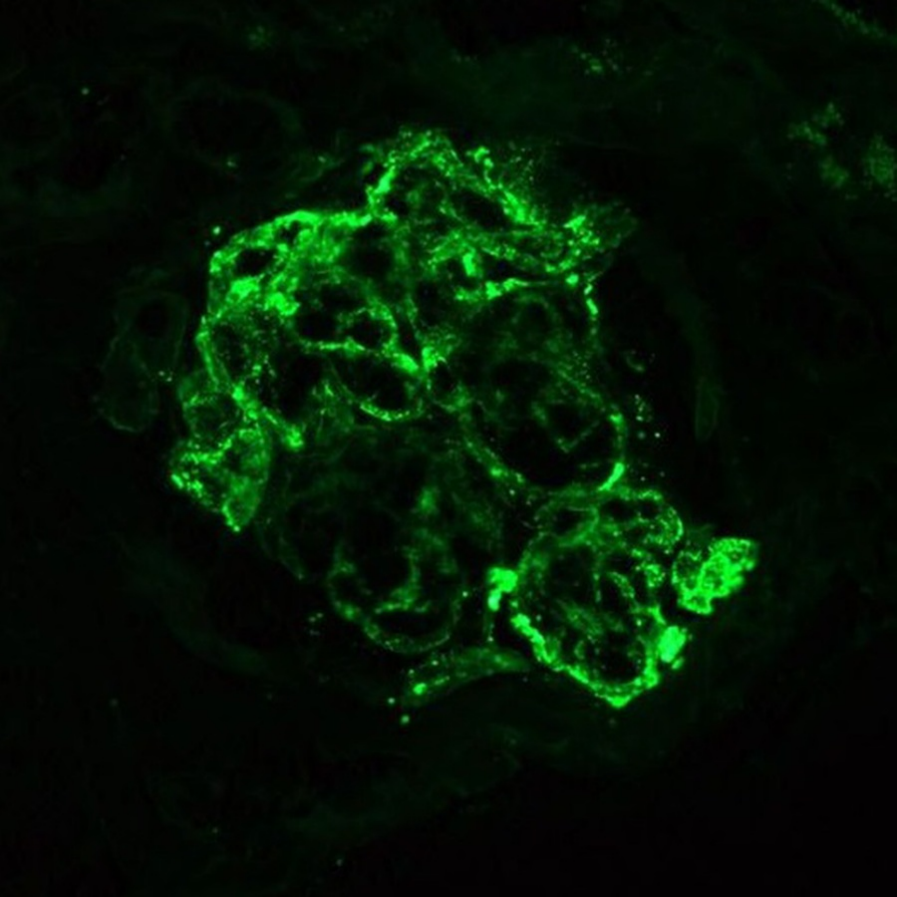
Immunofluorescent stain on kidney biopsy.

The findings of the renal biopsy were consistent with HSP, with proliferative and crescentic IgA-dominant glomerulonephritis [Figure 2]. Of the seven glomeruli viewed on light microscopy, four contained epithelial crescents; however, there were no globally sclerosed glomeruli. Immunofluorescence microscopy showed two glomeruli with immunoglobulin G (IgG), immunoglobulin M (IgM), IgA, C3, C4, C1q, albumin, fibrinogen, and kappa and lambda light chains [Figure 3]. All glomeruli showed diffuse endocapillary proliferation and influx of inflammatory cells, lymphocytes, and neutrophils.

With the definitive diagnosis of HSP, he was initiated on immunosuppressive therapy with cyclophosphamide 1000 mg initially, followed by a repeat dose in two weeks and then monthly for six months thereafter. His steroid therapy with methylprednisolone at 60 mg daily was continued.

Clinically, however, his GI involvement (continued hematochezia, hematemesis, diffuse abdominal pain) and renal function (worsening creatinine reaching a peak of 5.37 mg/dL) were not improving, and he developed new areas of rash over his limbs. Thus, plasmapheresis with cryoprecipitate replacement after each session was started five days following the first dose of cyclophosphamide, alternating with the days of his hemodialysis regimen, for a total of eight sessions.

His gastrointestinal symptoms finally improved with the addition of plasmapheresis, with improved abdominal pain, resolution of bleeding, and improved oral intake. His renal function, however, showed no sign of recovery, and the patient was discharged after a total of eight sessions of plasmapheresis on an outpatient hemodialysis regimen.

## Discussion

Henoch-Schönlein purpura (HSP) usually presents with the classic tetrad of symptoms—purpuric rash, arthralgia, abdominal pain, and renal disease [1]. It is the most common vasculitis in children, with a clinical diagnosis usually sufficient, has a predictable course, and is usually self-limiting [3]. However, as in the case described above, HSP tends to be more severe, with more atypical presentations in adults, and a biopsy is usually required to confirm the diagnosis [3-4].

Renal involvement tends to be more frequent and carries a worse prognosis with a higher risk of progression to chronic kidney disease (CKD) in adults compared to children. With microscopic hematuria being the most common renal manifestation in children, adults show a higher rate of gross hematuria with nephritic and nephrotic features [3,5]. Gastrointestinal manifestations are seen in about half of all HSP patients and can range from the commonly seen abdominal pain, nausea, vomiting, to rarely more severe involvement including gastrointestinal hemorrhage, bowel ischemia and perforation [6-7]. The gastrointestinal manifestations are seen with equal frequency in both children and adults, but the severe involvement is much less frequent in adults [3-4].

Management of HSP in adults in general and HSP nephritis in particular remains controversial. Randomized control trials in this area are scarce and often inconclusive [5]. In children, the disease is usually self-limiting, and severe cases usually respond to steroids. Compared to this, it is seen that immunosuppressant treatment is administered more often in adults [3]. This occurs despite the lack of rigorous evidence. Small studies have compared steroids alone with steroids in combination with cyclophosphamide for HSP nephritis and showed no additional benefit. [8]. Similarly, a small case series identified patients with severe HSP nephritis who were treated with steroids in combination with plasmapheresis with mixed outcomes [9]. Knowledge about treatment of severe gastrointestinal manifestations come mainly from case reports and series in children, in which immunosuppression and plasmapheresis have been used in the event of steroid failure [10].

Overall, our adult patient shared the signs and symptoms of abdominal pain, purpuric rash, imaging showing GI inflammation, and renal injury with the other adult patients previously reported. However, the severe bowel pan-inflammation, periappendiceal involvement, and rapid renal failure truly made our patient unique. With such limited evidence of what constitutes an ideal management strategy in adults, we escalated therapy over time given lack of response with each previous strategy employed. The addition of plasmapheresis to cyclophosphamide and steroids made a dramatic improvement in our patient’s GI symptoms, even though there was no improvement in his renal function.

## Conclusions

Diagnosis and management of Henoch-Schönlein purpura (HSP) in adults can be challenging as it is rare, with a tendency for severe manifestations and rapid progression, and due to lack of rigorous data regarding management. As such, an early clinical suspicion and rapid escalation of therapy if no contraindications are present could result in favorable outcomes.

Further research is needed to define management strategies for HSP in adults. While various combinations of steroids, immunosuppressive agents, and plasmapheresis have been used with mixed results in small series, randomized control trials are needed to prove benefit.
